# In simulated data and health records, latent class analysis was the optimum multimorbidity clustering algorithm

**DOI:** 10.1016/j.jclinepi.2022.10.011

**Published:** 2022-10-11

**Authors:** Linda Nichols, Tom Taverner, Francesca Crowe, Sylvia Richardson, Christopher Yau, Steven Kiddle, Paul Kirk, Jessica Barrett, Krishnarajah Nirantharakumar, Simon Griffin, Duncan Edwards, Tom Marshall

**Affiliations:** a Department of Statistics, University of Warwick, Coventry, CV4 7AL, UK; b Institute of Applied Health Research, University of Birmingham, B15 2TT, UK; c Institute of Applied Health Research, University of Birmingham, B15 2TT, UK; d University of Cambridge, Cambridge Biomedical Campus, Cambridge, CB2 0SR, UK; e Nuffield Department of Women’s & Reproductive Health, University of Oxford, John Radcliffe Hospital, Oxford, OX3 9DU, UK; f Health Data Science, AstraZeneca, 1 Francis Crick Avenue, Cambridge, Biomedical Campus, Cambridge, CB2 0AA, UK; g University of Cambridge, Cambridge Biomedical Campus, Cambridge, CB2 0SR, UK; h Institute of Applied Health Research, University of Birmingham, B15 2TT, UK; i Strangeways Research Laboratory Worts Causeway Cambridge CB1 8RN, UK; j Strangeways Research Laboratory, Worts Causeway, Cambridge, CB1 8RN, UK; k Institute of Applied Health Research, University of Birmingham, B15 2TT, UK

**Keywords:** Multimorbidity, Clustering methods, Electronic medical records, Latent class analysis, Hierarchical cluster analysis, Multiple correspondence analysis, K-means1

## Abstract

**Background and Objectives:**

To investigate the reproducibility and validity of latent class analysis (LCA) and hierarchical cluster analysis (HCA), multiple correspondence analysis followed by k-means (MCA-kmeans) and k-means (kmeans) for multimorbidity clustering.

**Methods:**

We first investigated clustering algorithms in simulated datasets with 26 diseases of varying prevalence in predetermined clusters, comparing the derived clusters to known clusters using the adjusted Rand Index (aRI). We then them investigated in the medical records of male patients, aged 65 to 84 years from 50 UK general practices, with 49 long-term health conditions. We compared within cluster morbidity profiles using the Pearson correlation coefficient and assessed cluster stability was in 400 bootstrap samples.

**Results:**

In the simulated datasets, the closest agreement (largest aRI) to known clusters was with LCA and then MCA-kmeans algorithms. In the medical records dataset, all four algorithms identified one cluster of 20–25% of the dataset with about 82% of the same patients across all four algorithms. LCA and MCA-kmeans both found a second cluster of 7% of the dataset. Other clusters were found by only one algorithm. LCA and MCA-kmeans clustering gave the most similar partitioning (aRI 0.54).

**Conclusion:**

LCA achieved higher aRI than other clustering algorithms.

## Introduction

Multimorbidity is the coexistence of two or more longterm health conditions [1]. Co-occurrence of diseases where all patients have a particular index condition (such as diabetes) is generally referred to as comorbidity [2]. Multimorbidity is becoming more important with an aging population and is linked to socioeconomic deprivation [3—5]. Multimorbidity can be understood in terms of its consequences and causes [6]. The consequences include increased complexity of clinical management, a high treatment burden, altered prognosis, and increased health care resource use, particularly when associated with functional impairment [7—10]. Particular combinations of diseases are more strongly associated with healthcare resource use and prognosis than the number of co-occurring diseases [11,12]. Resource use varies with different multimorbidity clusters and is modified by sociodemographic and household factors [13]. Current health services, clinical specialities, guidelines, quality improvement strategies, and quality of care metrics often reflect a single disease paradigm [14—17].

Multimorbidity is not a single entity, there are many different groups of co-occurring diseases. Some co-occur by chance, others because of common origins. To understand the causes of multimorbidity or to develop services for multimorbidity needs an understanding of which diseases tend to cluster. Due to the variety of ways in which potential combinations of diseases can be modeled, previously reported multimorbidity clusters have varied with different analytic methods [18—20]. In a typical problem requiring use of cluster algorithms, one would not know the true clusters. To understand how different clustering methods might perform in real health data we therefore need to investigate their performance in a simulation study, where the true clusters are known.

A systematic review of multimorbidity clustering studies identified four clustering algorithms used: exploratory factor analysis, cluster analysis of diseases, cluster analysis of people, and latent class analysis [21]. Two disease clusters (mental health conditions and cardiometabolic conditions) were identified consistently across all four algorithms and three further disease clusters by most clustering algorithms. However, few studies used more than one method, making it more difficult to directly compare the reproducibility of methods than if they had been applied to the same dataset.

In this paper we identify a number of methods used to group patients into clusters based on the combinations of multiple long-term health conditions in large datasets. We have applied these methods to investigate their reproducibility and validity, first in a large simulated dataset and then in a dataset of people with multiple long-term health conditions derived from electronic primary care records.

## 2 Methods

### 2.1 Identification of clustering methods

Methods commonly used to identify clusters of patients with similar multimorbid conditions were identified from two recent systematic reviews on clustering methods [20,21]. We selected the most frequently used methods for clustering patients (rather than diseases), those most applicable to binary data and which would scale for use on large datasets.

Four clustering algorithms were selected: latent class analysis (LCA) and hierarchical cluster analysis (HCA), as these methods were most frequently used in current multimorbidity research when clustering patients rather than diseases, and multiple correspondence analysis followed by k-means (MCA-kmeans) and k-means (kmeans), as these methods were applicable to binary data and scaled for use with large datasets.

Latent class analysis is a model-based clustering approach that derives clusters using a probabilistic model that describes the distribution of the data as opposed to determining clusters based on a chosen distance measure. In latent class analysis, posterior probabilities of cluster membership are assigned to each individual based on the estimated model parameters and their observed scores. This allows for each individual to be allocated to the appropriate latent class based on their probability of membership and from this, the risk of mortality by cluster can be estimated [22].

Hierarchical cluster analysis begins by calculating the distance between each pair of individuals using an appropriate distance measure. HCA can be applied agglomeratively (the algorithm starts with each individual as a single element cluster; at each iteration the two most similar clusters are merged, based on a linkage method, until all individuals are in one large cluster) or divisively (the algorithm starts with one cluster containing all individuals; the most heterogeneous cluster is split to form two clusters, until all individuals are single element clusters). We applied only agglomerative HCA. HCA can be visualized using a dendrogram which shows how clusters are merged or split and may indicate where the dendrogram can be cut to give an appropriate number of clusters. We used asymmetric binary distance, defined as the proportion of long-term health conditions where only one of the pair had the condition divided by the number of long-term health conditions where at least one of the pair had condition (1 - (A⋂B)/(A⋃B)). The linkage method was Ward’s minimum variance; at each iteration this merges the pair of clusters with the smallest between cluster variance. HCA allocates individuals to a single cluster and does not require prespecification of the number of clusters.

K-means is an iterative clustering method which partitions a dataset into k nonoverlapping clusters, allocating individuals to only one cluster. The algorithm begins by randomly selecting k observations (without replacement) as initial cluster centroids. The squared Euclidean distance between each remaining observation and each cluster centroid is calculated, the observation allocated to the closest cluster, and the cluster centroid is recalculated. This process is repeated until there is no further movement between clusters. K-means clustering is generally used for clustering continuous variables as it uses Euclidean distance to determine the distance between data points and cluster centers, however, it can be used with binary data [23].

Multiple correspondence analysis followed by k-means (MCA-kmeans) is a two-step approach which applies multiple correspondence analysis (MCA) to categorical data to reduce dimensions, followed by a k-means algorithm to define clusters [24].

### 2.2 Performance in a simulated dataset with known clusters

Clustering algorithms were first investigated in a simulated dataset with predetermined clusters using the adjusted Rand Index to compare the allocation of patients to clusters made by each algorithm to the known clustering of patients in the simulated dataset. The adjusted Rand Index is a widely used measure of cluster similarity which takes into account grouping by chance and produces a value from 0 to 1 (complete agreement) [25]. Most clustering algorithms require the user to specify the number of clusters in the data. For the simulated dataset, the number of clusters was known.

### 2.3 Generation of the simulated dataset

As we don’t have access to a true source of clustering, our simulated dataset is a simplified representation of disease clusters, with synthetic parameters matching our expectations of disease clusters in primary care records and the range of clustering parameters in studies reviewed by Ng [20]. Where possible, synthetic cluster data had parameters matched to a range seen corresponding to our model disease set (see below), or to approximate the distribution of clusters we expect based on clinical expertise. The mean prevalence within each cluster of diseases was varied over a range covering 1.5—90%, compared to an overall prevalence of any disease of 8.9% for our cardiometabolic data set. The number of diseases per simulated cluster was set around 5 (95% interval 2—10), corresponding to a medically expected range of expected diseases per cluster [3—11]. For intracluster disease correlations, we assumed for simplicity that they were identical and allowed them to vary as part of the sensitivity analysis. Within our dataset of primary care records, we observed (Pearson) correlations ranging between diseases from —0.2 to 0.8 [26]. Where parameters were harder to estimate such as “noise” (observations of a disease in a patient who is not a member of the cluster containing that disease) or the number of patients not in any cluster, these were varied as part of the sensitivity analysis.

For generating groups of correlated, simulated disease clusters, we used a multinomial probit model, in which the binary disease status is determined by a latent, multivariate-normal distributed variate. We generated a simulated 26-disease (denoted A-Z), multiple disease cluster dataset in three steps. The number of clusters of diseases was denoted K and the number of patients was *N*, resulting in an *N* × 26 matrix of disease observations. Each of the *N* patients was assigned as a member of one of the K disease clusters. The probability of a particular patient being assigned to a particular cluster was either distributed with an exponential, random weight over clusters 1, 2, K (with parameter lambda = 1), or uniformly (balanced).

1. Within each of the K disease clusters, the number of the 26 diseases within that cluster was determined by the maximum of (a) a Poisson distributed random variable with mean value 5, (b) the numerical value 2. This parameter choice was set to reflect our belief that in the range 2–10 conditions would be observed within a disease cluster. We either allowed overlap of a disease between clusters (e.g., disease A could occur in disease cluster 1 and 2) or not. In the case where no overlap was allowed, the assignment of diseases to disease clusters was performed sequentially, until none were left. Where overlap of clusters was allowed, assignments took place independently (i.i.d.)
2. For each patient in each of the K disease clusters we generated simulated observations of the 26 diseases using the multinomial probit model with a 26 × 26 correlation matrix. For a cluster k, we generated intradisease correlations for each of the D_k diseases by setting the off-diagonals of the correlation matrix between each of the D_k corresponding diseases to a prespecified (positive) correlation coefficient ρ. For example, the disease cluster number 1 may contain diseases {A, B, C} with an interdisease correlation between each disease of ρ = 0.2.
3. Uncorrelated, background noisy observations were added to each of the 26 columns of the resulting N x 26 matrix. The probability of each noise observation was allowed to vary to allow us to test resistance of cluster discovery to uncertain observation.

For each set of parameters we created 1,000 simulated datasets. Each clustering algorithm was applied to the simulated dataset a) including all observations and b) including only observations with two or more long-term health conditions present, this analysis intending to demonstrate the requirement for multimorbidity (presence of two or more conditions). The adjusted Rand Index was calculated, comparing the simulated “known” clusters with the clustering allocation found by the algorithm. Median, lower, and upper quartiles of the adjusted Rand Index were taken from the distribution of values from the individual simulations.

Parameters of the simulated dataset were varied to investigate the effect of correlation between diseases in the cluster, the prevalence of noise (within each cluster, the prevalence of diseases not allocated to the cluster), and the prevalence of diseases in each cluster; in each of these scenarios the dataset contained three clusters. When examining the effect of varying prevalence we tested disease prevalences between 1.5% and 90% in order to give an extreme example with defined clusters. In addition, we examined the effect of varying the number of clusters the algorithm was asked to find, using a simulated dataset with four clusters so that the effect of specifying fewer clusters could be examined. In simulated datasets, diseases were allowed to occur in more than one cluster and within cluster disease prevalence and noise prevalence was constant.

### 2.4 Investigation in primary care records

IQVIA Medical Research Data UK (IMRD UK) contains longitudinal primary care records for around 6% of the population from practices around the UK. The database has been shown to be representative of the UK population in terms of demography, prevalence of long-term conditions, and mortality [27]. Collection of data in IMRD was approved by the NHS South East Multi-Centre Research Ethics Committee (MREC) in 2003. We obtained approval to conduct this analysis from the Scientific Review Committee (reference number: 21SRC055).

A random sample of 50 practices from IMRD UK was selected and male patients, aged 65 to 84 and registered for at least 12 months on 1st January 2017 were included in the primary care sample. We chose this group as an exemplar as clustering may be gender and age-specific, and we wished to have a relatively homogenous group of patients in terms of multimorbidity. Long-term health conditions were defined as the presence (coded as a binary variable) of any of 49 conditions (listed in Table 1) recorded on or before 1st January 2017 and only conditions with at least 1% prevalence were considered, resulting in a similar list of conditions to that used in other analyses [3]. Patients with at least two long-term health conditions were included in the analysis.

We used plots of Bayesian Information Criterion (BIC), sample size adjusted BIC and entropy, applied to latent class analysis, to determine the optimal number of clusters for two to eight clusters. With the exception of HCA, each clustering algorithm used in the analysis was applied directly to the primary care dataset. As HCA is computationally intensive, it does not scale well to large datasets, therefore we used a hybrid method of applying k-means clustering to the data (specifying 50 clusters) then applied HCA to the resulting cluster centroids [28]. For LCA, patients were assigned to the cluster with the highest posterior probability; other algorithms assign patients to a cluster without giving the probability of membership for each cluster. All of the clustering algorithms assigned patients to nonoverlapping clusters, that is a patient could only be assigned to one cluster. As we clustered patients rather than conditions, it was possible for a condition to belong in more than one cluster. Clusters were named using the top three conditions with the greatest difference in within-cluster prevalence compared with prevalence in the full dataset.

To investigate whether clustering algorithms identified similar clusters we compared within cluster morbidity profiles (the proportion of patients with each disease in each cluster) from each pair of methods using Pearson correlation coefficient (PCC), high values indicating clusters with similar disease profiles [29]. Each cluster identified by an algorithm was matched to a cluster identified by a different algorithm, with the highest PCC. To ensure that a cluster could be matched to only one other cluster from an alternative algorithm, we found the pair of clusters with the highest PCC, these two clusters were excluded from further matching, then found the next highest PCC from the remaining clusters, and so on. Correlation coefficients greater than 0.5 were considered to indicate a similar cluster.

To assess the stability of clusters according to variations in the dataset (correlation between diseases, background noise and prevalence of disease), we selected 400 bootstrap samples from the original data and applied the clustering algorithms to each. Within a single clustering algorithm, each cluster in the bootstrap sample was matched with a cluster in the original data using the matching process described above. Mean and standard deviation of PCC (for PCC > 0.5) was calculated for the most similar cluster over all the bootstrap samples.

Bubble plots of exclusivity and observed/expected ratio (O/E ratio) were created to investigate the profile of all diseases within each cluster. Exclusivity was defined as the number of patients with the disease in the cluster divided by the total number of participants with the disease; larger bubbles indicate that a greater proportion of patients with a disease are present in a cluster. O/E ratio was calculated as the prevalence of a given disease within a cluster divided by its prevalence in the overall population; larger bubbles indicate that the prevalence of disease in the cluster is greater than the total population.

The adjusted Rand Index (aRI) was used to assess the similarity of partitioning between pairs of algorithms. We also examined whether the same patients were allocated to clusters with similar disease profiles. All analysis was undertaken in R [30].

## 3 Results

### 3.1 Simulated dataset

**Fig. 1:**
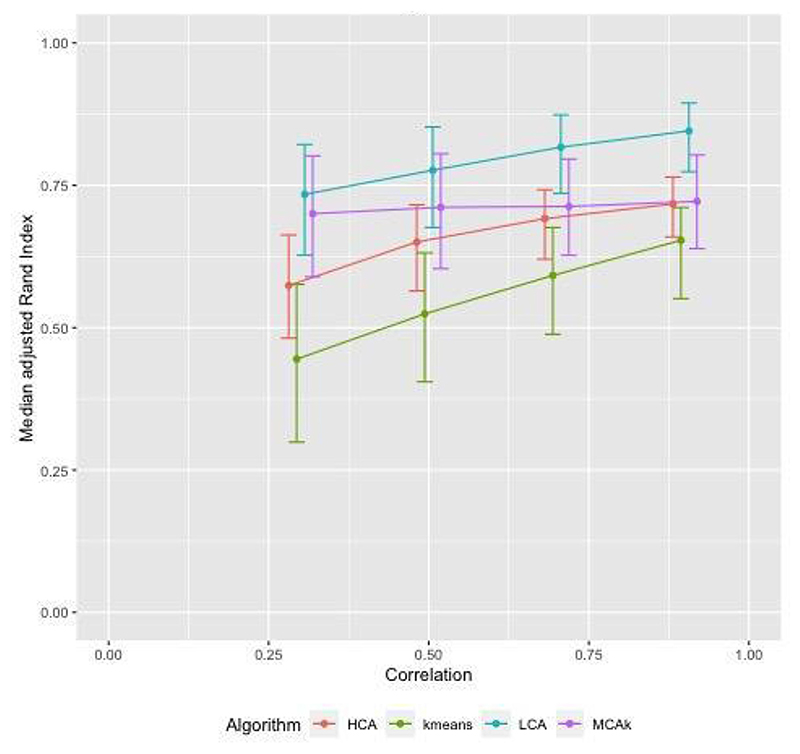
Simulated dataset of patients with two or more conditions in 3 clusters, within cluster disease prevalence approximately 15%, noise approximately 0.5%, overlap of diseases between clusters: examining the effect of varying correlation of diseases within a cluster. Error bars show interquartile range (IQR).

**Fig. 2:**
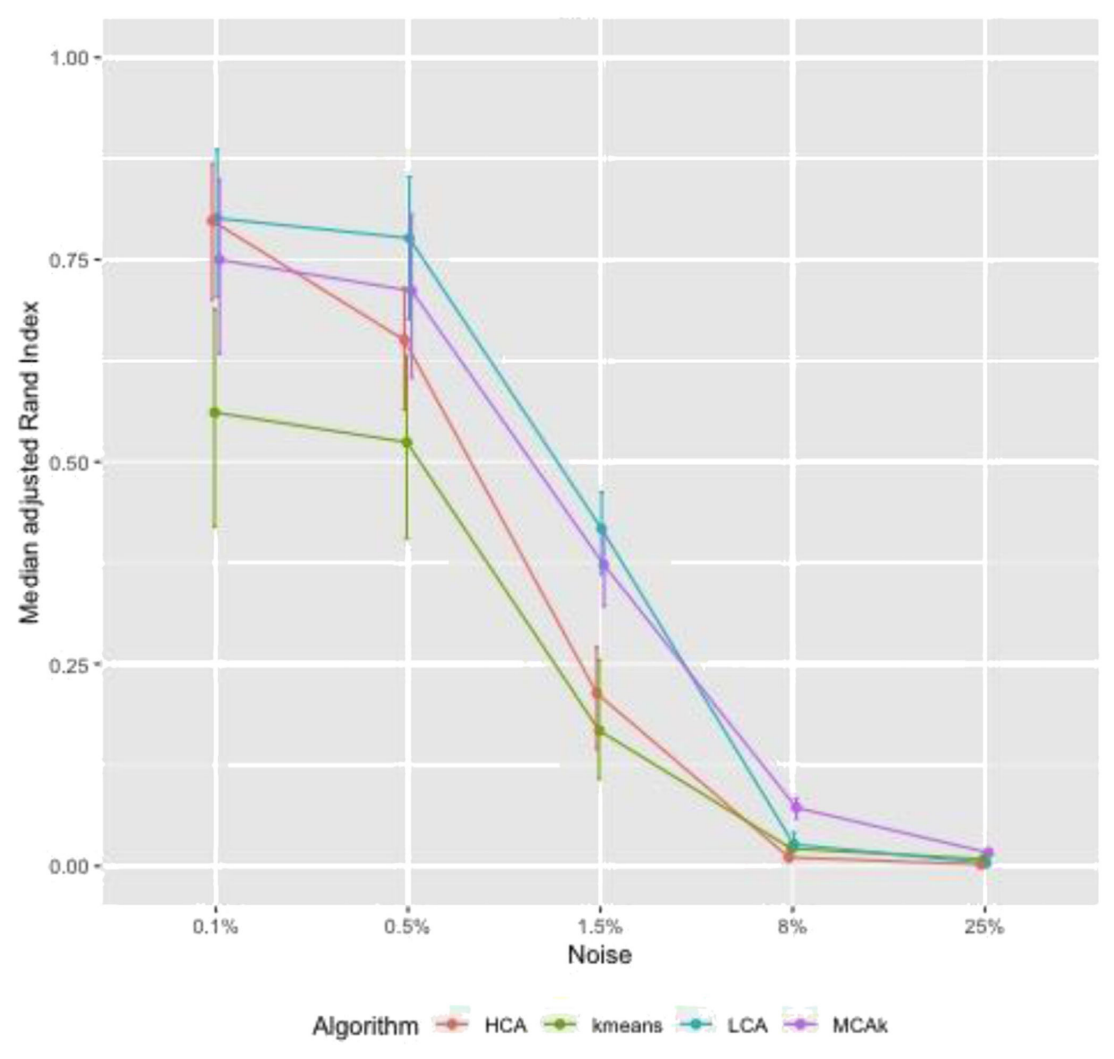
Simulated dataset of patients with two or more conditions in 3 clusters, within cluster disease prevalence approximately 15%, correlation = 0.5, overlap of diseases between clusters: examining the effect of varying the amount of noise.

**Fig. 3:**
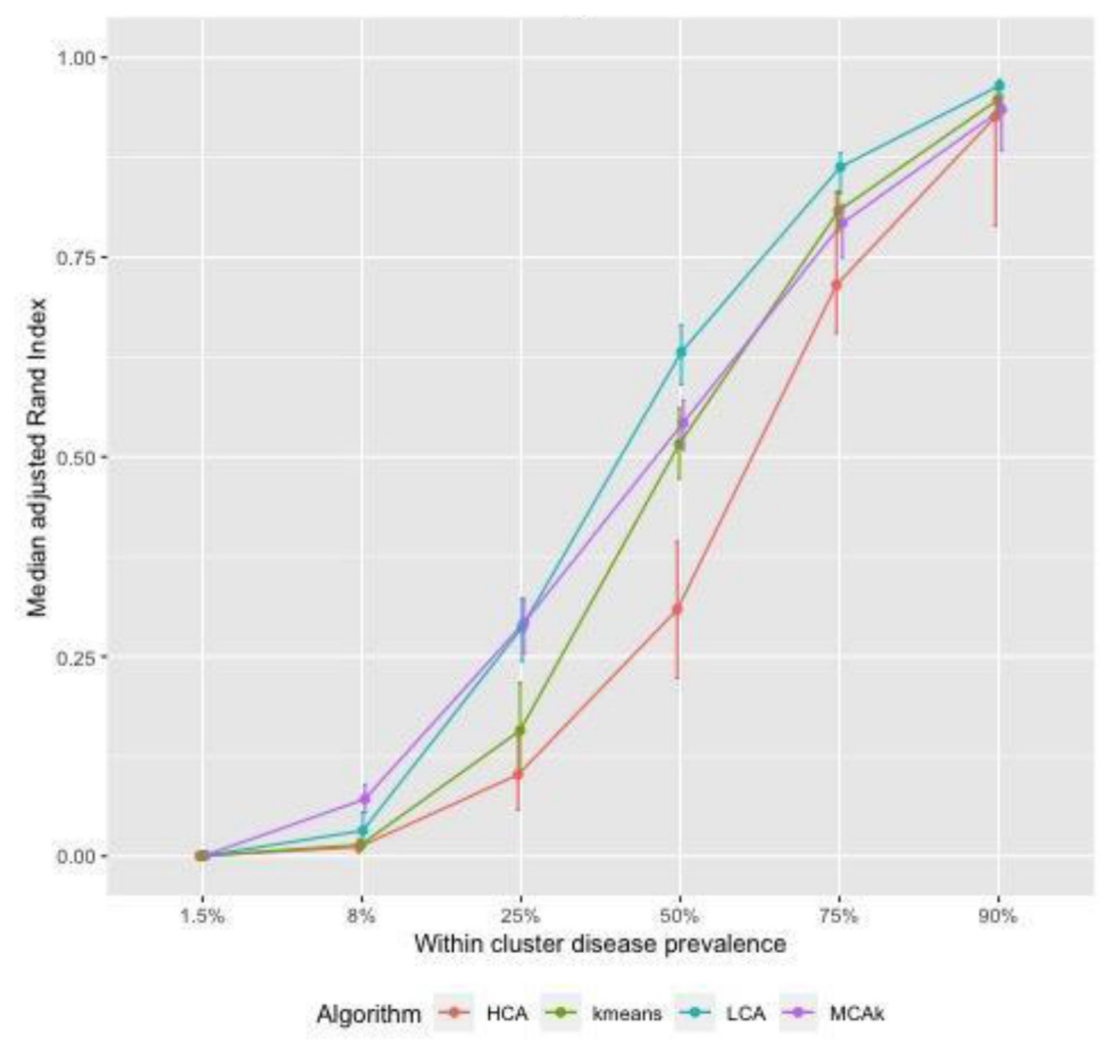
Simulated dataset of patients with two or more conditions in 3 clusters, noise approximately 4%, correlation = 0.5, overlap of diseases between clusters: examining the effect of varying within cluster prevalence of disease.

**Fig. 4:**
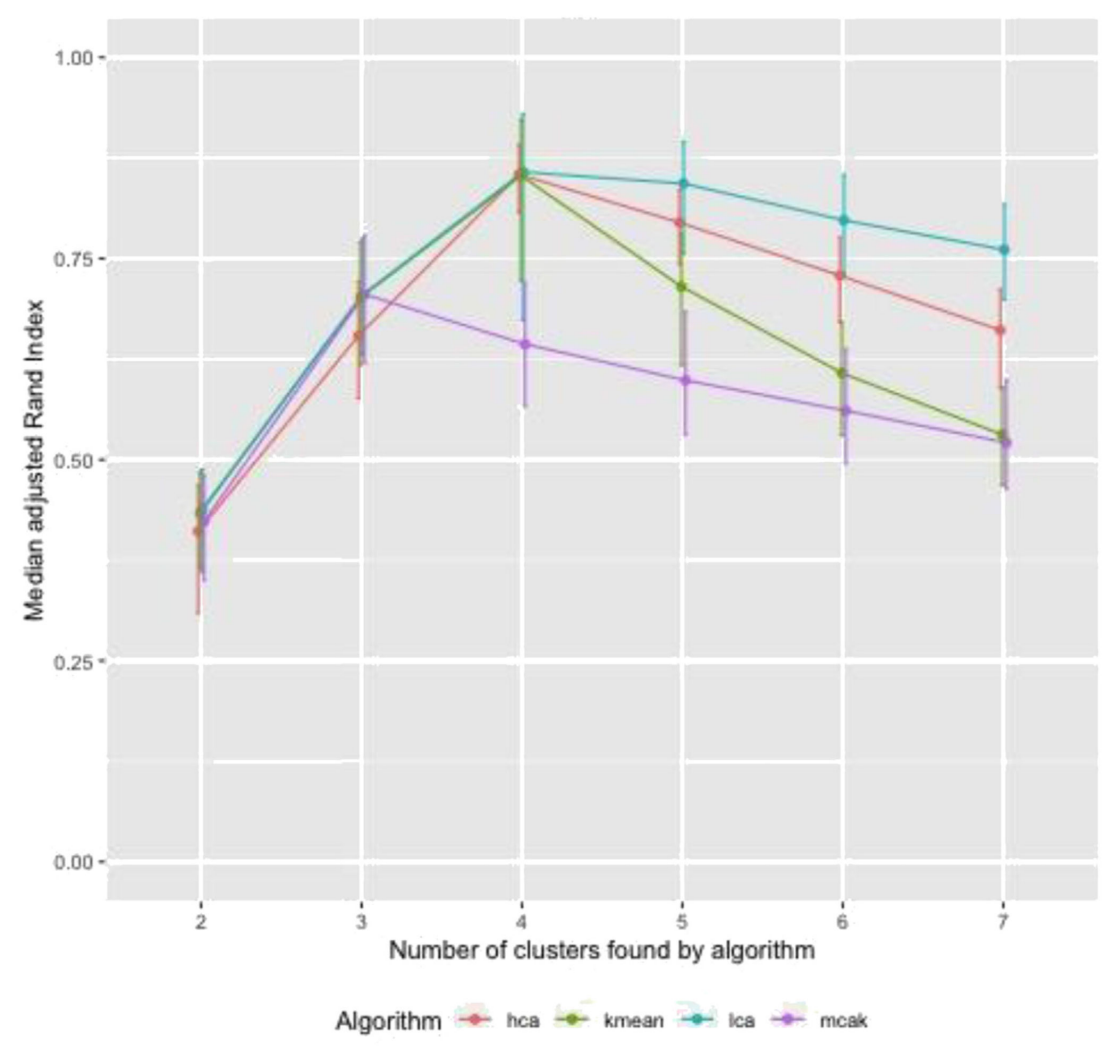
Simulated dataset of patients with two or more conditions in 4 clusters, within cluster disease prevalence approximately 24%, noise approximately 0.5%, correlation = 0.5, overlap of diseases between clusters: examining the effect of varying the number of clusters algorithm is asked to find.

Figure 1 shows adjusted Rand Index (aRI) as the correlation between diseases in the cluster was varied between 0.3 and 0.9. For HCA and kmeans aRI increased by approximately 0.2 as correlation changed from 0.3 to 0.9, for the other algorithms the increase was smaller. The aRI decreased as the prevalence of diseases not in a cluster (noise) increased. As the prevalence of noise increased to reach the prevalence of diseases in the cluster, aRI was close to zero (Fig. 2). There was a positive association between aRI and the prevalence of disease (Fig. 3) with aRI approaching 1 as the prevalence reached 75%. The aRI remained constant as the number of clusters increased (Fig. 4). With the exception of MCA-kmeans, all of the algorithms gave the highest aRI for four clusters (the true number of clusters in the simulated dataset). MCA-kmeans found the highest aRI for three clusters.

In order to consider whether cluster techniques should be applied to a restricted sample to include only those who are multimorbid or to all patients, clustering algorithms were also applied to entire simulated datasets, including non-multimorbid patients (those with none or only one condition) (Appendix 1). Adjusted Rand Index values were close to zero for most scenarios, the exception being simulations with high prevalence of disease; when disease prevalence was high the proportion of non-multimorbid patients was low resulting in similar aRI’s in the multimorbid sample and the population.

Across the scenarios tested by the simulated dataset, LCA and MCA-kmeans algorithms gave the closest agreement (largest aRI) between known cluster allocation and those identified by the algorithm, although there was overlap in the distribution of aRI by algorithm.

### 3.2 IMRD UK data

There were 23,251 males aged 65 to 84 years with two or more long-term health conditions in the analysis dataset. Table 1 shows the prevalence of conditions in these patients. The median number of long-term health conditions was 4 (IQR 3—6) and over half (57%) of the study population had hypertension. The optimal number of clusters identified was four. A cluster with lead conditions of erectile dysfunction (ED), diabetes, and hypertension or ischemic heart disease (IHD) was found across all algorithms; this cluster accounted for 20%—25% of the study population. A cluster of patients with heart conditions (including heart failure, IHD, atrial fibrillation, other heart disease, and valvular disease) was identified by LCA and MCA-kmeans algorithms, comprising approximately 7% of the study population (Table 2). Other clusters found by only one of the clustering algorithms included a group of patients with high prevalence of peripheral vascular disease and aortic aneurysm (found using latent class analysis) and a cluster with respiratory conditions (asthma, COPD and bronchiectasis) found by Kmeans-HCA algorithm (Appendix 2). The bubble plots (Appendix 2) shows that each algorithm found at least one cluster with high levels of exclusivity for most diseases, indicating that no particular group of conditions characterized the cluster, it was a “catch-all” group. Latent class analysis and MCA-kmeans clustering gave the most similar partitioning of patients, with adjusted Rand Index of 0.54, all of the other algorithm pairings had aRI between 0.2 and 0.3.

As the diabetes-ED-hypertension cluster was found by all algorithms we investigated whether this cluster found by different methods contained the same patients. This cluster contained approximately 5,000 patients, depending on the algorithm used, 4,120 (85% of the 4,869 patients in this cluster identified by LCA) patients were found to be in this cluster across the four methods. Similarly, the heart disease cluster found by LCA and MCA-kmeans algorithms had 1,247 patients in common (58% of the 2,161 patients in this cluster identified by LCA).

For the clusters found by LCA there was a corresponding cluster with a similar morbidity profile found by the MCA-kmeans algorithm (Table 3). However, for all other algorithm pairings one of the four clusters was not matched to a cluster with a similar morbidity profile: for kmeans the cancer-depression-COPD cluster was not matched; for kmeans-HCA algorithm the asthma-COPD-rhinitis cluster was unmatched; and for the heart failure-IHD-atrial fibrillation cluster found by MCA-kmeans none of the clusters generated by kmeans-HCA and kmeans had similar morbidity profiles (Table 3).

When comparing within each method, in all bootstrap samples the clusters found by LCA, MCA-kmeans and kmeans algorithms could be matched with a similar cluster in the original sample. For kmeans-HCA, the cancer-depression-eczema cluster and the asthma-COPD-rhinitis were most sensitive to variations in the data, with 96% and 47% respectively of the bootstrap samples having a cluster with a similar morbidity profile (Table 4).

## 4 Discussion

Our analysis investigated the stability and reproducibility of clusters identified using four different clustering algorithms in a range of simulated datasets with a known number of clusters. We then investigated the replicability of clusters using the four methods in a dataset derived from primary care records where the number of true clusters was unknown.

In a simulated dataset the aRI was influenced modestly by the degree of within-cluster correlation. However increasing the amount of noise or reducing the disease prevalence both markedly reduced the aRI. Under most scenarios LCA achieved higher aRI than other clustering algorithms (MCA-kmeans, kmeans, and kmeans-HCA). Findings on simulated datasets suggest that there may be a threshold of disease prevalence, below which, the clustering methods do not perform very well, and similarly a threshold for the amount of noise in the data. However, it is difficult to give an estimate of where these thresholds might lie due to the difficulties in creating simulated data which reflects actual data.

When seeking four clusters in a dataset derived from primary care records, all the clustering algorithms (LCA, MCA-kmeans, kmeans and kmeans-HCA) identified one similar large cluster (diabetes-ED-hypertension) including mainly the same patients. This cluster included three of the conditions with the highest prevalence in the whole dataset (diabetes, ED and hypertension), which may have been grouped due to their prevalence, as the simulations found when disease prevalence was higher all algorithms were better at finding clusters, or the cluster may have occurred as a result of clinical recording. LCA and MCA-kmeans algorithms both identified one smaller cluster (heart disease cluster) including mostly the same patients. Other clusters were identified by only one of the methods and each method identified a non-specific “catch all” cluster. LCA and MCA-kmeans were the methods most consistent with each other. Within-method repeatability, assessed by taking bootstrap samples of the data, was high for LCA, MCA-kmeans and kmeans but repeated analyses using kmeans-HCA sometimes identified clusters with different disease profiles.

In datasets where the true characteristics of the population are known, LCA may perform better over other methods. The observation that kmeans-HCA identifies less repeatable clusters than other methods suggests it may not be ideal for clustering analysis of long-term health conditions. Because inclusion of patients without multimorbidity increases the noise and markedly reduces reproducibility, it is likely to be more useful to exclude patients without comorbidities from analysis datasets.

### 4.1 Comparison with other studies

One study observed some agreement in clusters identified using HCA and exploratory factor analysis in a large dataset of primary care records, but did not investigate k-means, LCA or MCA [18]. In terms of the clusters found in the primary care data, other studies have found clusters of cardiometabolic disease and mental health conditions [21]; only the cardiometabolic cluster was found in our data, and not by all algorithms. Roso-Llorach et al. [18] found a cluster which included diabetes and hypertensive disease in male patients, which may be similar to our diabetes-ED-hypertension cluster.

### 4.2 Strengths and limitations

We investigated a range of clustering algorithms used in multimorbidity studies although not all clustering algorithms were investigated. Our approach makes use of both simulated data and simplified electronic health records data. A key limitation is the extent to which the analysis datasets are generalizable to real world data. The consistency of findings across both the simulated data and the primary care dataset increases confidence in the findings.

A limitation of applying the findings from our simulated datasets is that these may not reflect the complexities of observed data. For example, there were a larger number of diseases in the primary care dataset than in simulations, also correlation between diseases was assumed to be constant for all diseases in the simulated clusters, while in the primary care data correlation between diseases was generally very low; only two pairs of conditions had correlation greater than 0.5 (aortic aneurysm and PVD; and diabetes and ED).

There are wider limitations to clustering algorithms using routine data sources. Our analyses use binary disease categories whereas in reality, most diseases are categories imposed on a continuous distribution of clinical features. This means there is potential for chance misclassification. Systematic misclassification may occur if propensity to assign a diagnosis to clinical features is associated with the presence of other diagnoses. It may also occur if recording of clinical features is affected by ascertainment bias, e.g., routine management of some long-term health conditions includes undertaking diagnostic tests or actively asking about specific symptoms (e.g., asking about erectile dysfunction at annual diabetes reviews). Because clusters may be artifacts of the process of data gathering and recording, detailed knowledge of these processes also greatly assist interpretation of clustering.

## Supplementary Material

Supplementary File 1

Supplementary File 2

## Figures and Tables

**Table 1:** List of conditions and prevalence in males aged 65-84 yr

Condition	Short name	Prevalence (%)
Hypertension	Hyp	56.7
Erectile dysfunction	ED	36.0
Osteoarthritis	OA	27.8
Diabetes	Diab	24.6
Ischemic heart disease	IHD	24.5
Deafness	Deaf	22.2
All cancer	Can	21.7
Benign prostatic hypertrophy	BPH	19.4
Eczema	Ecz	18.4
Chronic kidney disease	CKD	15.4
Depression	Dep	14.8
Asthma	Asth	14.4
Gout	Gout	14.3
Atrial fibrillation	AF	13.4
Cataract	Cat	12.7
Stroke/TIA	Stroke	11.6
COPD	COPD	11.0
Diverticulitis	Div	10.6
Rhinitis/conjunctivitis	Rhin	10.1
Anxiety	Anx	9.7
Peripheral vascular disease	PVD	8.1
Peptic ulcer	Pep	7.1
Heart failure	HF	6.9
Psoriasis	Psor	6.5
Sinusitis	Sinus	5.6
Hypothyroid	Hypothy	5.5
Glaucoma	Glau	4.7
Irritable bowel syndrome	IBS	4.7
Heart valve disease	Valve	4.3
Migraine	Mig	4.2
Alcohol/substance misuse	Addict	4.0
Autoimmune disease of connective tissue	Tissue	4.0
Venous thromboembolism	VTE	3.4
Obstructive sleep apnoea	OSA	3.0
Osteoporosis	Osteo	2.7
Aortic aneurysm	Aneu	2.6
Autoimmune disease of bowel	Bowel	2.6
Alzheimers/dementia	Dem	2.5
Other autoimmune disease	Auto.oth	2.4
Epilepsy	Epi	2.2
Pulmonary embolism	PE	2.1
Age-related macular degeneration	AMD	1.8
Blindness	Blind	1.8
Chronic liver disease	Liver	1.6
Serious mental illness	SMI	1.3
Bronchiectasis	Bronc	1.3
Parkinson’s disease	Park	1.2
Other heart disease	Oth.heart	1.2
Hyperthyroid	Hyperthy	1.1

**Table 2:** Clusters found in population of males aged 65-84 yr

Top 3 conditions (prevalence in cluster, %)			Number of patients (% of total)
Latent class analysis			
Diabetes (100%)	Erectile dysfunction (89%)	Hypertension (69%)	4,869 (21)
Eczema (19%)	Cancer (22%)	IBS (5%)	15,257 (66)
Heart failure (55%)	Atrial fibrillation (61%)	IHD (68%)	2,161 (9)
PVD (100%)	Aortic aneurysm (62%)	IHD (49%)	964 (4)
MCA-kmeans			
Rhinitis/conjunctivitis (11%)	Eczema (19%)	IBS (5%)	11,563 (50)
Diabetes (83%)	Erectile dysfunction (91%)	Hypertension (73%)	5,503 (24)
IHD (41%)	Atrial fibrillation (28%	COPD (25%)	4,463 (19)
Heart failure (54%)	IHD (71%)	Atrial fibrillation (55%)	1,722 (7)
Kmeans			
Hypertension (100%)	CKD (18%)	Gout (16%)	7,002 (30)
Diabetes (100%)	Erectile dysfunction (99%)	Hypertension (69%)	5,005 (22)
Osteoarthritis (100%)	BPH (25%)	Deafness (25%)	5,099 (22)
Cancer (24%)	Depression (16%)	COPD (12%)	6,145 (26)
Kmeans-HCA			
Hypertension (83%)	Gout (23%)	IHD (32%)	7,346 (32)
Cancer (33%)	Depression (18%)	Eczema (26%)	8,222 (35)
Erectile dysfunction (96%)	Diabetes (83%)	IHD (61%)	6,142 (26)
Asthma (100%)	COPD (46%)	Rhinitis/conjunctivitis (29%)	1,541 (7)

**Table 3:** Pearson correlation coefficient to compare within-cluster morbidity profile across algorithms

Clustering algorithm and clusters identified	MCA-kmeans				Kmeans				Kmeans-HCA			
	Rhin-Ecz-IBS	Diab-ED-Hyp	IHD-AF-COPD	HF-IHD-AF	Hyp-CKD-Gout	Diab-ED-Hyp	OA-BPH-Deaf	Ca-Dep-COPD	Hyp-Gout-IHD	Ca-Dep-Ecz	ED-Diab-IHD	Asth-COPD-Rhin
Latent class analysis												
Diab-ED-Hyp	0.51	**0.99**	0.38	0.66	0.51	**1.00**	0.39	0.24	0.51	0.41	**0.99**	0.23
Ecz-Ca-IBS	**0.99**	0.57	0.87	0.60	**0.85**	0.52	0.80	0.47	0.89	**0.90**	0.55	0.53
HF-AF-IHD	0.58	0.59	0.82	**0.95**	0.65	0.61	**0.56**	0.46	**0.77**	0.56	0.62	0.37
PVD-Aneu-IHD	0.40	0.37	**0.59**	0.63	0.47	0.38	0.38	0.27	0.57	0.37	0.38	0.21
MCA-kmeans												
Rhin-Ecz-IBS					**0.83**	0.50	0.79	0.44	0.86	**0.91**	0.53	0.51
Diab-ED-Hyp					0.57	**0.99**	0.42	0.25	0.57	0.44	**0.99**	0.24
IHD-AF-COPD					0.75	0.41	**0.74**	0.57	**0.86**	0.81	0.44	0.56
HF-IHD-AF					0.63	0.71	0.52	0.41	0.75	0.49	0.72	0.33
Kmeans												
Hyp-CKD-Gout									**0.91**	0.66	0.51	0.42
Diab-ED-Hyp									0.52	0.41	**1.00**	0.24
OA-BPH-Deaf									0.71	**0.76**	0.42	0.38
Ca-Dep-COPD									0.25	0.57	0.33	0.43

The most similar cluster, with the highest Pearson correlation coefficeint (PCC) is shown in bold. Clusters with a PCC < 0.5 are considered not similar.

**Table 4:** Mean Pearson correlation coefficient comparing within-cluster morbidity profile between bootstrapped and original data (where PCC >0.5). Based on 400 bootstrap samples

Clustering algorithm and clusters identified	Mean PCC (SD)	Number of samples with PCC >0.5
Latent class analysis		
Diab-ED-Hyp	0.9995 (0.0004)	400
Ecz-Ca-IBS	0.9973 (0.0033)	400
HF-AF-IHD	0.9880 (0.0437)	400
PVD-Aneu-IHD	0.6613 (0.1532)	400
MCA-kmeans		
Rhin-Ecz-IBS	0.9976 (0.0083)	400
Diab-ED-Hyp	0.9992 (0.0016)	400
IHD-AF-COPD	0.9757 (0.0903)	399
HF-IHD-AF	0.9938 (0.0216)	400
Kmeans		
Hyp-CKD-Gout	0.9996 (0.0028)	400
Diab-ED-Hyp	0.9998 (0.0001)	400
OA-BPH-Deaf	0.9986 (0.0231)	400
Ca-Dep-COPD	0.9982 (0.0142)	400
Kmeans-HCA		
Hyp-Gout-IHD	0.8961 (0.0605)	400
Ca-Dep-Ecz	0.8134 (0.0989)	385
ED-Diab-IHD	0.9922 (0.0045)	400
Asth-COPD-Rhin	0.8126 (0.1499)	186

## What is new?

### Key findings

- We directly compared the stability of four clustering algorithms for multimorbidity clustering in a simulated dataset and in a dataset of electronic primary care records

### What this adds to what was known?

- Latent Class Analysis (LCA) and Multiple Correspondence analysis followed by kmeans (MCA-kMeans) algorithms gave the closest agreement to known clusters and the most similar partitioning

### What is the implication and what should change now?

- Individuals with a single long-term health condition should be excluded when undertaking clustering analysis
- LCA and MCA-kMeans are preferred methods for clustering analysis when investigating multimorbidity in large datasets
