# Supplementary material for: In simulated data and health records, latent class analysis was the optimum multimorbidity clustering algorithm
Source: J Clin Epidemiol. Author manuscript; Available in PMC 2022 Nov 27. (PMC7613854; doi:10.1016/j.jclinepi.2022.10.011)
Supplement: Supplementary File 1 [file EMS157097-supplement-Supplementary_File_1.pdf]

Patient allocation to disease clusters

Let the patient numbered i be denoted P_i. For the imbalanced scenario with K clusters, the (un-normalized) weighting for patient i being allocated to disease group k, w_k^i is uniform over the patients and distributed exponentially over the clusters:

$$Pr(w_k = x) \sim exp(-x)$$

The patient is allocated to disease group k with probability

$$Pr(P_i(G = k)) = w_k / \Sigma_{j=1}^{K} w_j$$

For the balanced scenario, the probability of allocation to any disease cluster is equal to $1/K$:

$$Pr(P_i(G = k)) = 1/K$$

Disease allocation to disease clusters

Let the disease cluster with number k be denoted DC_k and numbered 1 through K. Let the k'th disease cluster DC_k denote n_k diseases $(d_i, \dots, d_{n_k})$.

The number of diseases allocated to disease cluster k, n_k is Poisson distributed, with floor set to 2 and rate λ equal to 5.0:

$Pr(n_k = m) \ \sim \mathbb{1}_{m \geq 2} \mathrm{Pn}(\lambda; m)$ (up to normalization)

and for each cluster k, the n_k diseases $d_1, \dots, d_{n_k}$ are drawn with uniform probability, without replacement, from N_D diseases $D_1, \dots, D_N$ (numbered 1 through 25). For disease cluster k, DC_k, with n_k diseases in cluster k:

$$Pr(DC_k(d_1 = D_i, \dots, d_{n_k} = D_k); i < \dots < k, k \leq N) = n_k!(N - n_k)!/N! = 1/\binom{N}{n_k}$$

Simulation of disease presence and absence from clusters

Let the observed presence or absence of disease d in patient i be denoted Y_i^d (0,1). We model the relationship between simulated disease presence and absence for patient i allocated to k'th disease group DC_k, as a multinomial probit:

$$Pr(Y_i^{sim, DC_k} = 1 | X^{DC_k}) \sim \Phi(X^{DC_k}, \Omega^{DC_k})$$

where Φ is the standard multivariate normal and X^{DC_k}, Ω^{DC_k} are a (n_k dimensional) latent variable and n_k x n_k latent correlation matrix controlling the disease indicators. The value of the latent probit mean for disease group k, X^{DC_k} is set at a uniform value (-0.70).

As we might expect some correlation structure within a disease group, we set all off-diagonal correlations within Ω^{DC_k} to some uniform positive value $0 \leq \rho \leq 0.7$, which we can vary.

Addition of background noise to observations

We model the presence of uncorrelated background noise which could contaminate our cluster observations by adding a background value via an uncorrelated latent probit mean for the N_D diseases in the N_P x D dimensional observation matrix Y. This is done by adding a latent value of the noise floor M, set to a uniform value over all disease for simplicity:

$$Pr(Y_i^{spurious,d} = 1) \sim \Phi(M^{N_D})$$

$$Y_i^{obs,d} = \max(Y_i^{spurious,d}, Y_i^{sim,d})$$

In [ ]:
