# Supplementary material for: In simulated data and health records, latent class analysis was the optimum multimorbidity clustering algorithm
Source: J Clin Epidemiol. Author manuscript; Available in PMC 2022 Nov 27. (PMC7613854; doi:10.1016/j.jclinepi.2022.10.011)

### Appendix 1. Plots of simulated data, algorithms applied to full dataset including patients with zero or one condition

Figure S 1: Simulated dataset of 6000 patients in 3 clusters, 2000 patients not in a cluster, prevalence approximately 15%, noise approximately 0.5%, overlap of diseases between clusters: examining effect of varying correlation of diseases within a cluster. Error bars show interquartile range (IQR)


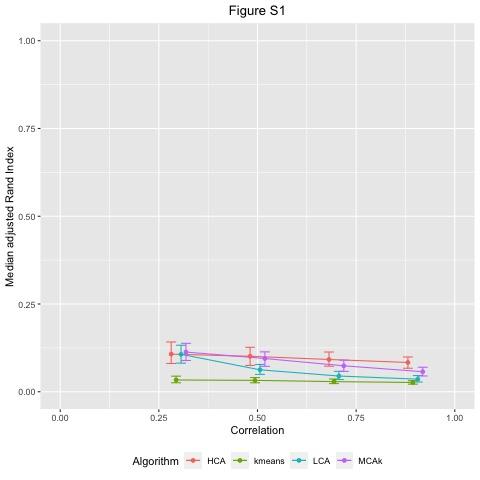


Figure S 2: Simulated dataset of 6000 patients in 3 clusters, 2000 patients not in a cluster, prevalence approximately 15%, correlation=0.5, overlap of diseases between clusters: examining effect of varying amount of noise.


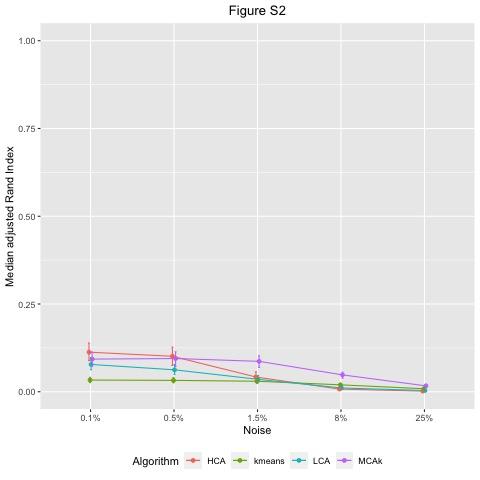


Figure S 3: Simulated dataset of 6000 patients in 3 clusters, 2000 patients not in a cluster, noise approximately 4%, correlation=0.5,overlap of diseases between clusters: examining effect of varying the prevalence of disease.


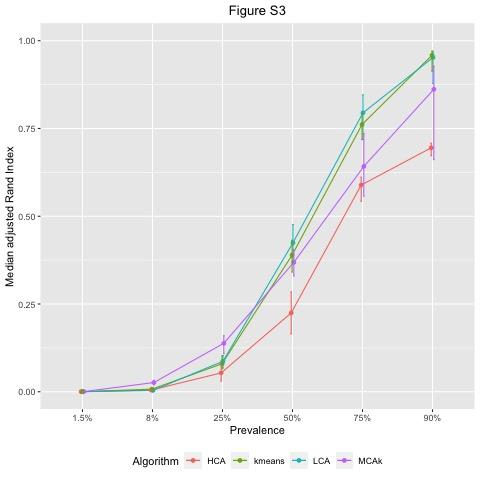


Figure S 4Simulated dataset of 2000 patients in 4 clusters, prevalence approximately 24%, noise approximately 0.5%, correlation=0.5, overlap of diseases between clusters: examining effect of varying the number of clusters algorithm is asked to find.


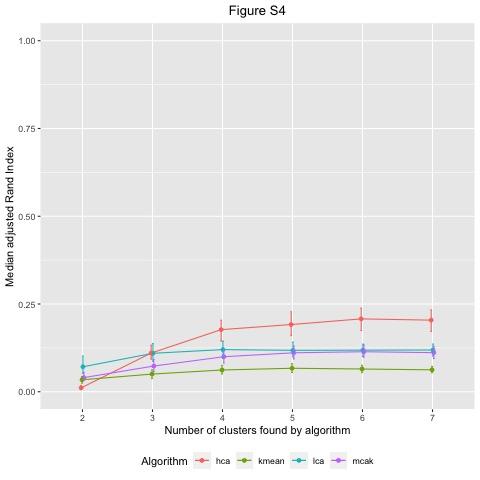


### Appendix 2. Bubble plots of exclusivity and O/E ratio

Bubble plot 1: exclusivity of clusters found by latent class analysis


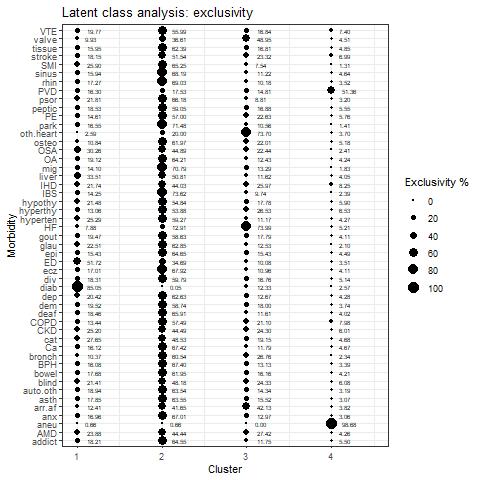


Bubble plot 2: O/E ratio of clusters found by latent class analysis


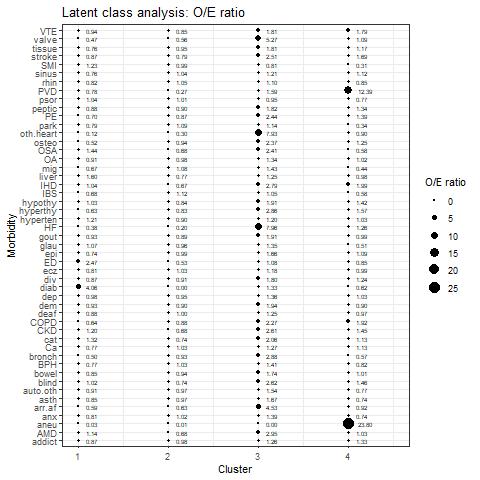


Bubble plot 3: exclusivity of clusters found by MCA-kmeans clustering


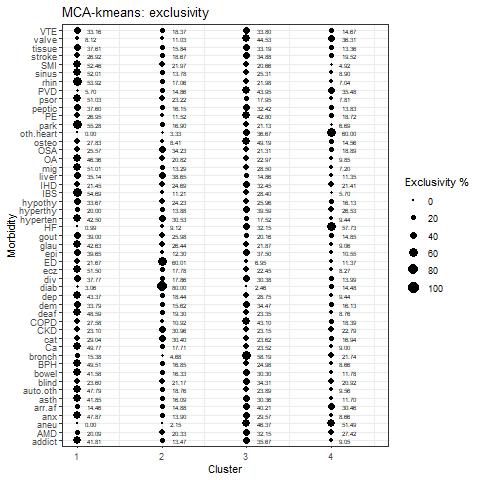


Bubble plot 4: O/E ratio of clusters found by MCA-kmeans clustering


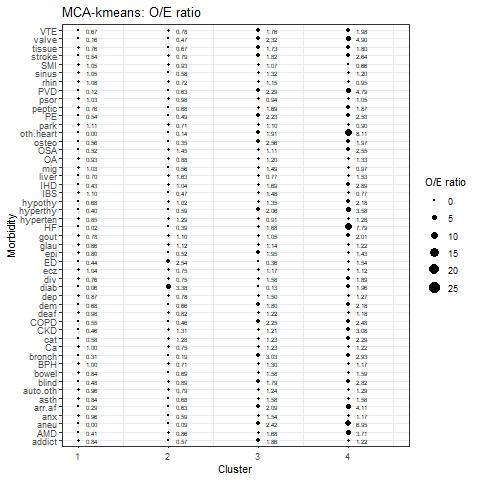


Bubble plot 5: exclusivity of clusters found by kmeans clustering


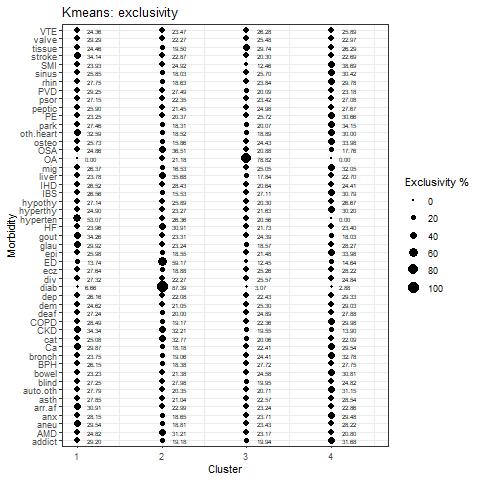


Bubble plot 6: O/E ratio of clusters found by kmeans clustering


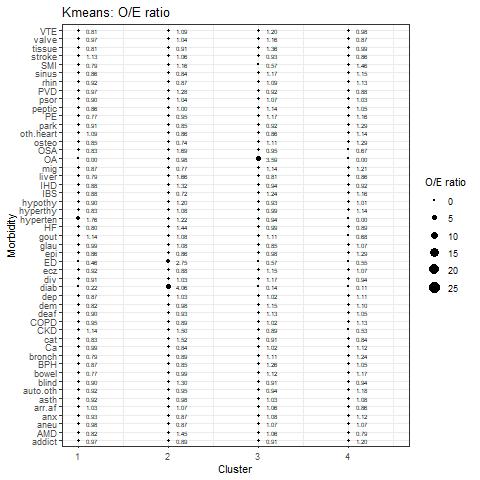


Bubble plot 7: exclusivity with kmeans-HCA clustering


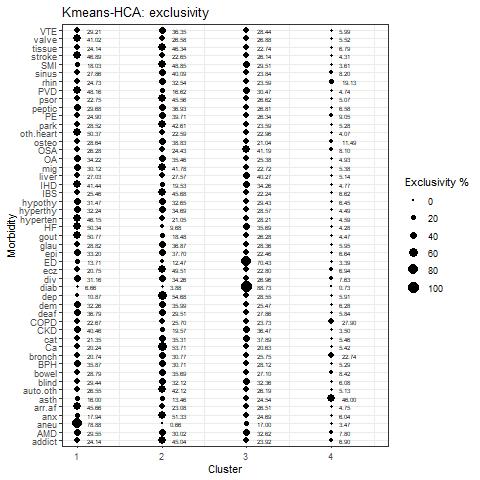


Bubble plot 8: O/E ratio with kmeans-HCA clustering


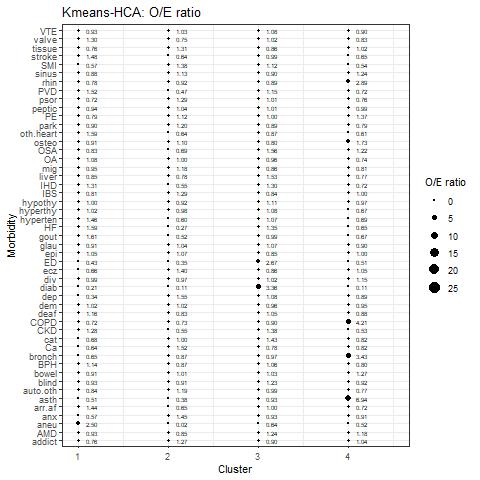

Supplement: Supplementary File 2 [file EMS157097-supplement-Supplementary_File_2.docx]
